# Characterization of *Bordetella pertussis* Strains Isolated from India

**DOI:** 10.3390/pathogens11070794

**Published:** 2022-07-14

**Authors:** Shweta Alai, Manish Gautam, Sonali Palkar, Jitendra Oswal, Sunil Gairola, Dhiraj P. Dhotre

**Affiliations:** 1 Department of Health Sciences, Symbiosis International University, Pune 412115, India; shweta.alai236@gmail.com; 2 Clinical Bioanalytics Laboratory, Serum Institute of India Pvt. Ltd., Pune 411028, India; m.gautam@seruminstitute.com (M.G.); sunil.gairola@seruminstitute.com (S.G.); 3 Bharati Vidyapeeth Medical College, Pune 411043, India; palkarsh@gmail.com (S.P.); jsoswal@gmail.com (J.O.); 4 National Cell Centre for Science (NCCS), Pune 411007, India

**Keywords:** genetic divergence, whooping cough, developing country, allele, genotype, phylogeny, Oxford sequencing, vaccines

## Abstract

Despite high level vaccination and the availability of two different types of vaccines, whole cell (wP) and acellular vaccines (aP), the resurgence of pertussis has been reported in many countries. Antigenic variation within circulating and vaccine strains is the most documented reason reported for the resurgence of pertussis. Research on genetic divergence among circulating and vaccine strains has largely been reported in countries using aP vaccines. There are inadequate data available for antigenic variation in *B. pertussis* from wP-using countries. India has used wP for more than 40 years in their primary immunization program. The present study reports five clinical isolates of *B. pertussis* from samples of pediatric patients with pertussis symptoms observed in India. Genotypic and phenotypic characterization of clinical isolates were performed by serotyping, genotyping, whole genome analyses and comparative genomics. All clinical isolates showed serotype 1, 2 and 3 based on the presence of fimbriae 2 and 3. Genotyping showed genetic similarities in allele types for five aP genes within vaccine strains and clinical isolates reported from India. The presence of the ptxP3 genotype was observed in two out of five clinical isolates. Whole-genome sequencing was performed for clinical isolates using the hybrid strategy of combining Illumina (short reads) and oxford nanopore (long reads) sequencing strategies. Clinical isolates (*n* = 5) and vaccine strains (*n* = 7) genomes of *B. pertussis* from India were compared with 744 *B. pertussis* closed genomes available in the public databases. The phylogenomic comparison of *B. pertussis* genomes reported from India will be advantageous in better understanding pertussis resurgence reported globally with respect to pathogen adaptation.

## 1. Introduction

Whooping cough, also called as pertussis, is caused by the Gram negative bacterial pathogen *Bordetella pertussis* [1]. Among other members of the Bordetella species such as *Bordetella parapertussis*, *Bordetella Bronchiseptica* and *Bordetella Holmesii*, which shows similar respiratory infections in humans, *Bordetella pertussis* is a major cause of pertussis disease. Pertussis is a vaccine preventable disease. Whole cell pertussis vaccine (wP) used as a component of a combined diphtheria, tetanus, and pertussis vaccine (DTwP) and acellular pertussis vaccines (DTaP) are employed globally as part of an expanded program of immunization in children [2]. High vaccination coverage with two distinct types of vaccines led to a significant reduction in morbidity and mortality due to pertussis globally.

Many countries that switched to aP due to fear associated with the side effects of wP, such as reactogenicity, have reported a resurgence in the pertussis disease [3]. Acellular pertussis vaccines (DTaP) comprise purified inactivated virulence proteins of *Bordetella pertussis*, including pertussis toxin (PT), pertactin (PRN), fimbriae (FIM 2,3) and filamentous hemagglutinin (FHA), in different compositions [4,5]. One of the major hypotheses reported for resurgence is antigenic divergence within genes mostly encoding aP components [4]. The antigenic variation hypothesized due to escape from immunity owing to limited inclusion *B. pertussis* antigens in the aP vaccine composition and also due to differential immune response generated by different vaccines, i.e., wP, appears to be mediated largely by Th1 cells, whereas aP induces Th2 and Th17 responses [6]. aP induces immune response only against five antigens, which suggests selective pressure induced by acellular vaccine associated with pathogen adaptation. Therefore, the introduction of aP may, in due course, result in new adaptations in the *B. pertussis* population [7]. Antigenic divergence is suggested by the dominance of new types of alleles for the five genes in vaccine and circulating strains. These current strains can be less susceptible to vaccine-induced immunity and thus cause vaccine escape strains of *B. pertussis*. The gene type for five genes dominating in vaccine strains, referred as *PtxP1*, *PtxA*2, *fim*2-1, *fim3*-1 and prn1, were replaced in the current *B. pertussis* population. In vaccine-type alleles of *B. pertussis* toxin A observed as *ptxA*2 and *ptxA*4, these allele types replaced with *ptxA*1 allele predominate *B. pertussis* circulating population. As per Bart et al., (2016) all pre-vaccination strains embraced with prn1 allele, which relates to the vaccine-type allele (6). Similarly, the proportion of prn1 allele decreased and prn3 and prn2 strains increased. The vaccine-type alleles of *B. pertussis* fim2-1 and *fim3*-1 were found in all vaccine strains, subsequently replaced by fim2-2 and *fim3*-2 in a circulating population [8]. The bacterial adaptation in circulating *B. pertussis* strains was also noted concerning the emergence of antigen deficient strains [9]. The new circulating strains deficient for *B. pertussis* antigens such as prn, FHA, and Ptx were reported in several countries. Pertactin deficient strains were reported initially in Philadelphia (USA) and subsequently found in many countries using aP vaccines such as Japan, France, Finland, Australia, and Italy [10]. Altogether, along with waning immunity and increased surveillance, the antigenic variation among vaccine clinical strains observed in the *B. pertussis* may describe the persistence of pertussis disease, despite the high levels of immunization coverage. Reports on antigenic divergence and pertussis resurgence are mostly available from countries using acellular vaccines [11,12,13,14,15]. Such data are missing from countries using DTwP. Developing countries such as India have been using DTwP vaccines in their national immunization programs for many years [16]. Very few reports of resurgence and antigenic variation are reported from developing countries including India.

Due to inadequate documentation, epidemiology data for pertussis from India is limited. As per WHO estimates, incidences of pertussis in India declined sharply after the introduction of vaccination in 1960. India reported 200,932 cases and 106 deaths in the year 1987 with a mortality rate of <0.001%. A gradual decrease was reported in pertussis incidence between 1981 and 1995. Although pertussis cases have fluctuated in recent years, increases were observed in 1997–2016 with cases ranging from 217,371 to 37,274. In 2009, a rise in incidences of pertussis was reported with 60,385 cases (WHO estimates, 2020). Data on exact health burden and incidence of pertussis in the Pune region are rare and available figures lack specificity. Therefore, genetic characterization studies on circulating isolates from developing countries such as India will be important to better understand antigenic divergence between vaccine and clinical strains. We previously reported genetic characterization data of pertussis vaccine strains used by manufactures in the production of wP strains [17]. We also reported comparative genomics of two available *B. pertussis* clinical isolates BPD1 and BPD2 from India [18]. We report here five clinical isolates of *Bordetella pertussis* recently collected from India (S1, S2, S3, S4, and S5) with genotypic and phenotypic characterization, and comparative genomics with vaccine strains and global reported genomes of *B. pertussis*. The study included isolation and characterization of *B. pertussis* (S1, S2, S3, S4, S5) from clinical samples received from patients hospitalized with clinical features of pertussis, which is the highest number of isolates characterized and complete genome sequences deposited in the database so far from India. 

In this study, isolates were characterized using polymerase chain reaction (PCR), multilocus antigen sequence typing (genotyping), serotyping, and comparative genomics of clinical isolates with vaccine strains and globally reported isolates. Genotyping of pathogenicity associated genes is necessary to study antigenic divergence within *B. pertussis* strains [19]. Serotyping is one of the conventional methods used to characterize *B. pertussis* isolates serotypes [19]. Three serotypes 1, 2 and 3 were used to diagnose *B. pertussis* isolates, which is based on the presence of antigen Lipo-oligosaccharides (agglutinogen 1), fimbrae2 (fim2) and 3 (*fim3*). The serotypes seem to change within the population, therefore it is important to monitor dominant serotypes within vaccine and circulating strains [20,21]. In addition to the conventional PCR methods based on the two markers, IS481 and PtxP, consequently, we developed a 16 target multiplex conventional PCR, which includes species-specific gene targets, virulence genes and housekeeping genes of *B. pertussis* [22,23]. Here, we sequenced genomes of all five isolates using a hybrid strategy of combining long read using Oxford nanopore and short sequence reads using Illumina [24]. *B. pertussis* assemblies using such a hybrid strategy provide sequencing reads generally longer than the repeat elements, with the prospective to reveal genome features previously unnoticeable in multi-contig sequencing assemblies generated by short-read sequencing alone [25]. Complete genome sequences for all five isolates defined as S1, S2, S3, S4 and S5 was deposited in the public database. All *B. pertussis* genomes reported from India so far, vaccine strains (*n* = 6) and clinical isolates (*n* = 7) were compared with 744 whole genomes available in the public database to study the phylogenomic positioning.

## 2. Results

### 2.1. Isolate Characterization

*B. pertussis* (*n* = 5) isolates used in the present study are designated as S1, S2, S3, S4 and S5. The typical characteristics of the isolates and clinical samples are shown in Table 1. All five isolates were identified as a *B. pertussis* with colony characteristics as tiny, glistening, compact pearl-like colonies after 72 h of growth on BGA plates and were stained as Gram-negative cocci-shaped bacilli [26]. *B. pertussis* colonies show no growth on Mac-Conkeys agar plates and identified as oxidase positive in conventional biochemical tests [27]. All five isolates showed positive agglutination with *B. pertussis* specific polyclonal antibodies (NIBSC). Further isolates were confirmed as *B. pertussis* by matching the 16s rDNA sequences of ~1440 bp using a MicroSEQ™ Full Gene kit The MicroSeq, Applied Biosystems, Foster City, CA, USA) with known reference 16S region rDNA sequences in the MicroSEQ™ ID Analysis Software library (The MicroSeq, Applied Biosystems, Foster City, CA, USA) [28]. For all five isolates, sequence identity confirmed cultures of S1, S2, S3, S4 and S5 as *B. pertussis* with values in the range 99.94–99.99%, matching with the reference library. Similarly, at protein level using Maldi-ToF tool, all five isolates were confirmed as *B. pertussis* with organism best match against the reference library, with score values from 2.091–2.099, where the 2.000–3.000 score range suggests genus and highly probable species identification [29].

### 2.2. Fimbrial Serotyping

Fimbriae, which are considered as important antigens of *B. pertussis*, help in the attachment of the pathogen to the host tissues and are important for whole cell pertussis vaccines and a major component of acellular pertussis vaccines. *Bordetella pertussis* strains have both fim2 and *fim3* genes, although they may express either or both [30]. B. pertussis can be classified into different serotypes based on expression of fimbriae antigens. If the agglutination reaction was obtained with the *Fim*2 antibody, the *Fim*3 antibody, or both antibodies, the serotype was defined as *Fim*2, *Fim*3, or *Fim*2.3, respectively. *FimFim*All five isolates expressed both fim2 and *fim3* and showed agglutination with specific monoclonal antibodies. All five isolates S1, S2, S3, S4 and S5 showed all three antigens and thus fimbrial serotype 1, 2 and 3 (Table 1). Vaccine strains J445, J446, J447, J448 were serotype *Fim*2,3, whereas vaccine strain TohamaI was serotype *Fim*2.

### 2.3. Genotypic Analysis

**Figure 1:**
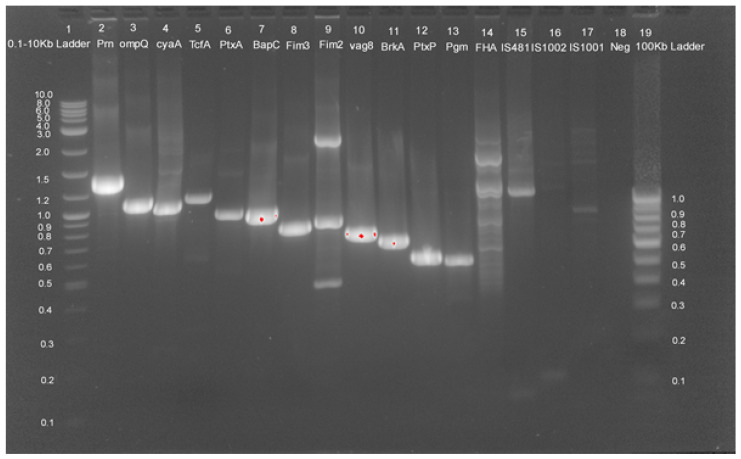
Agarose gel electrophoresis (2%) of PCR amplified products using virulence and species-specific PCR primer sets. Lanes 2–17 are examined for *B. pertussis* isolates. Lanes 2–17 from Isolate S1 for prn, ompQ, cyaA, TcfA, *PtxA*, BapC, *Fim*3, *Fim*2, vag8, BrkA, PtxP, Pgm, FHA, IS481, IS1002, IS1001, respectively. Lane 1: 0.1–10 kb DNA size marker, Lane 20: 100bp DNA size marker. Lane 18: Negative control.

Circulating *B. pertussis* strains showed signs of adaptive changes, with both antigenic divergent and antigen deficient isolates being recovered in recent years [11,12,30,31]. Multiplex PCR was used to detect virulence markers and species-specific genes in a single multiplex panel. The target panel was designed for twelve virulence genes in *B. pertussis* (prn, *ptxA*, ptxP, cyaA, tcfA, FHA, fim2, *fim3*, dnt, ompQ, brkA, bapC, vag8), the housekeeping gene phosphoglucomutase (pgm) of *B. pertussis* and species-specific genes (IS481, IS1001, IS1002) (Supplementary Table S1). The PCR results of all five isolates showed the presence of all twelve virulence markers and confirmed the identity with *B. pertussis*-specific housekeeping genes pgm and IS481, IS1002 (Figure 1).

### 2.4. Multilocus Antigen Sequence Typing (MAST)

Globally, single nucleotide variations have mainly been reported in five antigenic determinant genes as *ptxA*, prn, ptxP, *fim3*, fim2 [32]. All five genes were identified as genotypes for five isolates S1, S2, S3, S4 and S5 individually using sangers sequencing. The allele type identified for all five isolates are described in Table 2. The current dominant allele profile for all globally circulating isolates is *ptxA*1, prn2, ptxP3, *fim3*-2 and *fim*2-2 [33]. Changes in the allelic profile with the vaccine strains were reported from many countries during the resurgence of the disease [34,35]. Here, S1, S2, S5 isolates showed allelic profiles similar with the vaccine strains used in India and two isolates previously reported from India (Table 2) [36]. Most dominant circulating allele type for pertussis toxin, ptxP3 was identified in two isolates S3 and S4 used in this study and not observed in two other *B. pertussis* genomes present in the database from India [36,37].

### 2.5. Genome Organization 

**Figure 2:**
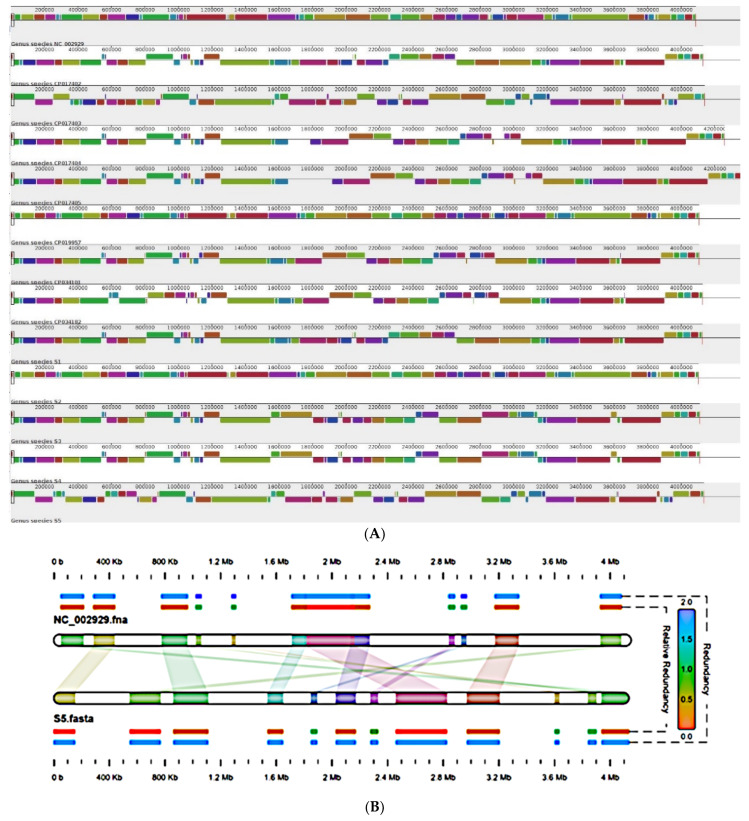
Whole genome alignment of *B. pertussis* genomes from India (**A**) Genome alignment of vaccine and clinical isolates. Homologous blocks are represented by the same colour and connecting lines show rearrangements within homologous blocks. (**B**) Inversion were analyzed using NC_002929 (Tohama I) as reference with clinical isolate S5.

**Figure 3:**
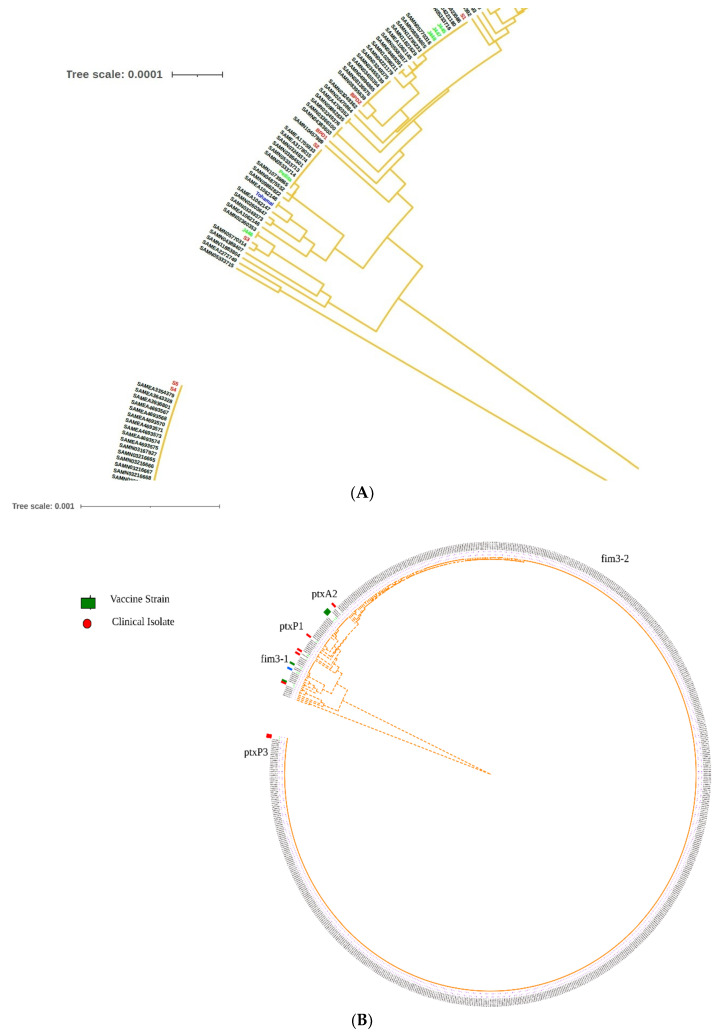
Global positioning of Indian *B. pertussis* genomes (**A**) Cut out section of phylogeny highlighting *B. pertussis* strains reported from India (**B**) Mash-based tree generated from whole genomes of all *B. pertussis* genomes using UPMGA highlighting sequenced types of isolates and vaccine strains from India S1, S2, S3, S4, S5. Green color indicate vaccine strains, red color indicate clinical isolates reported from India, Blue color indicate reference strain TohamaI.

For genome organization, whole genomes was aligned using the progressive mauve algorithm with default parameters and Tohama as a reference genome. The whole genome alignment of recent isolates S1, S2, S3, S4 and S5 of *B. pertussis* with all genomes available from India including vaccine reference strains and two previously reported clinical isolates (BPD1 and BPD2) was observed to study rearrangement such as inversion and deletion within the genomes in comparison with reference strain Tohama I [12,26,27,29]. The genome alignment of all recent isolates and previously characterized vaccine and clinical strains from India, belonging to fim2-1 and *fim3*-1 lineage, suggested that all *B. pertussis* genomes contain small scale structural rearrangements (Figure 2) [33,34]. Genomes of all thirteen *B. pertussis* strains suggested 261 homologues structures, including small inversions. According to genome organization, all thirteen *B. pertussis* genomes could be classified into two groups based on the closest similarity with Tohama I [33]. Cluster 1 showing similarity with TohamaI consisted of genomes Pelita, and S1, while all remaining ten genomes showed small scale inversion and few deletions compared to Tohama I [34]. In this second cluster, genomes can be again sub-dived into two groups consisting of S3 and S4 in one group. This separation was also verified by using phylogenetic tree with 772 *B. pertussis* genomes, available in the database from different countries (Figure 3). Region of differences, that RD3 (28.7 kb, spans genes BP0910A-BP0937) and RD10 (25.1 kb, spans BP1948-BP1968) was absent in S3 and S4 genomes within aligned genomes were observed [35]. Genome alignment of all thirteen genomes showed that genomes could be classified into three groups. Cluster one comprised genomes of *PtxP1* lineage such as J445, J447, J448, BPD1, BPD2, S1, S2, Pelita, which showed similarity concerning the presence of the RD3, RD5 and RD 10 region. Cluster 2 with PtxP3 lineage appeared to have lost a cluster of genes as RD3, RD5, RD10, which consisted of S3 and S4 isolates. S5 showed the maximum number of observations compared to Tohama I within region 1.7Mb to 2.4Mb (Figure 2B).

### 2.6. Global Positioning of B. pertussis Genomes Reported from India

To position the Indian (vaccine, reference and clinical isolates) *B. pertussis* genomes in a global context, we reconstructed mash-based phylogeny of 744 closed genomes available in the public database (as of 20 February 2021) using UPGMA tree algorithm with all genomes. The phylogenomic position of clinical and vaccine reference strains placed all genomes majorly in two different clusters due to *PtxP1* and PtxP3 allele (Figure 3). General genome characteristics are provided in Table 2. Vaccine strains J445, J446, J447, J448 and Pelita were classified into a single cluster with *PtxP1*, *fim3*-1 and fim2-1 alleles. In the same cluster, isolates S1, S2, S5 and BPD1, BPD2 were also observed. Isolates S3 and S4 comprised a separate cluster with strains similar to PtxP3 alleles. None of the isolate and vaccine reference strains were observed related to *fim3*-2 and fim2-2 cluster. (Figure 3). Vaccine strain J446 was slightly separated within *fim3*-1 cluster from all other genomes due to *ptxA*4/prn7 genotype. We also carried out the multilocus sequence typing (MLST) of all the publicly available isolates (*n* = 744) and compared the global position of Indian genomes based on sequence type in MLST analysis [37]. We observed only three genomes J446, Pelita and S2 belonging to ST 1, while all other genomes belonged to ST2 types, which are dominant in available genomes from different countries (Figure 3).

## 3. Discussion

To better understand the bacterial adaptation to vaccine-induced selection pressure on *Bordetella pertussis*, we needed to compare *B. Pertussis* population in countries where wP have been continuously used. India has been using whole-cell vaccines for immunization against pertussis for more than 40 years and continues to do so with a high vaccination coverage. Here, we report the very first studies on *B. pertussis* isolation and characterization reported from the Pune region within India, as a part of a pilot study. Clinical samples of pediatric patients with pertussis symptoms collected during 2016–2021. Thirty-four percent of the total collected samples proved to be Bordetella species by PCR targeting 16s multiplex panel, only twenty-four percent (five isolates) were culture positive. The initial detection of Bordetella positives through PCR varied the number of *B. pertussis* isolates actually cultured and may be not due to the difference in the sensitivity of the techniques only, but also due to decrease in bacterial viability. This may occur in a loss due to bacterial load, temperature, and duration during transport of clinical samples; antibiotic treatment was received before sample collection [38].

Initial detection of pertussis positive samples was performed using an in-house developed multiplex PCR panel including 16s bordetella gene targets. Traditional PCR assays target two major genes of *B. pertussis*: (1) the PT promoter region, and (2) insertion elements (IS481) [39]. IS481 is also present in *B. holmesii* and *B. bronchiseptica*. Thus, IS481 is not a species-specific target and can lead to false positive results for *B. pertussis.* The pertussis toxin is a major virulence factor of *B. pertussis,* whose genes are organized in operons and present in all three Bordetella, which is only expressed in *B. pertussis* and can be differentiated by a different melting temperature [40]. So, without post amplification analysis, a single copy target cannot differentiate between three species. Similarly, increasing cases of pertussis such as symptoms due to other members of the Bordetella species, namely, *B. Bronchispetica*, *B. holmesii*, need species-specific targets in the diagnostic tools. Pertussis vaccination does not provide cross protection against other species. Therefore, accurate diagnosis of causative bordetella is important to determine prophylactic treatment. In recent times with the adaption of an organism to vaccine-induced selection pressure, antigen deficient isolates have been found in many countries. Pertactin, FHA, TcfA deficient isolates were recently identified in many countries. It is important to update current diagnostic tests with new bordetella strains. We developed a multiplex PCR panel to detect virulence markers and species-specific genes in a single multiplex panel. The target panel was designed for twelve virulence genes in *B. pertussis* (*prn*, *ptxA*, *ptxP*, *cyaA*, *tcfA*, *FHA*, *fim2*, *fim3*, *dnt*, *ompQ*, *brkA*, *bapC*, *vag8*), the housekeeping gene phosphoglucomutase (pgm) of *B. pertussis* and species-specific genes (IS481, IS1001, IS1002). Further confirmation of the organism was carried out using biochemical methods, agglutination, 16s r-RNA sequencing and Maldi-ToF.

All isolates (S1, S2, S3, S4 and S5) were hemolytic and showed similar biochemical characteristics with the population. All isolates showed serotype 1, 2 and 3, which suggested properties of a good vaccine candidate as per WHO guidelines and recommendations for WCVs. In genotyping, we analyzed the allele profiles for five DTaP coding genes as per the recommended typing method of *B. pertussis*. We observed a common allele profile for clinical isolates as *ptxA*1/prn1/*fim3*-1/fim2-1. Divergence in pertussis toxin promoter region (ptxP) was observed in two clinical isolates S4 and S5. Vaccine type ptxPS1 allele was observed in three isolates (S1, S2, S3), whereas two isolates (S4, S5) showed non-vaccine genotype, i.e., ptxP3 [5,6,31]. We also compared clinical isolates with vaccine strains and seven 744 *B. pertussis* isolates. Our previous studies reported the genotype profile of vaccine strains used by a pertussis vaccine manufacturer from India, such as (J445:-ptxP1/*ptxA*2/prn1/fim2-1/*fim3*-1; J446:-ptxP2/*ptxA*4/prn7/fim2-2/*fim3*-1; J447 and J448:-ptxP1/*ptxA*1/prn1/fim2-1/*fim3*-1) [17,18]. Global positioning of strains reported from India was divided into two major groups, one *PtxP1* and a second cluster with the PtxP3 group.

Many studies suggested circulating isolates with the emergence of hypervirulent ptxP3 strains [41,42]. The ptxP3 strain has been proven to be more virulent, as suggested by increased pertussis toxin production, indicating an adaptive response to the selective pressure of high vaccine coverage [43]. The reemergence of ptxP3 strains were also reported by few countries using wP. Poland is the country with highest vaccination coverage, where WCV have been majorly used and completely shifted from *ptxA*2 to *ptxA*1 and ptxP1-ptxP3, prn1-prn2 and fim2-1 to fim2-2; alleles were reported in clinical isolates [44,45]. The wP vaccine has been used in Mexico for more than 50 years, where the appearance of ptxP3 strains started initially between 2006 and 2014 [46]. The predominant profile of circulating strains from Cambodia was ptxP3/*ptxA*1/prn2/*fim3*-1, where WCV was introduced in 1986 [47]. The characterization of isolates from Brazil, which has used WCV since 1977 in their primary immunization program, suggested a change in profiles from vaccine strains [48,49]. PtxP3 strains containing clinical isolates were also recently reported from this country. Pertussis vaccination in Iran was introduced in the 1950s with high coverage by wP vaccines, where allelic variation was observed between vaccine and clinical strains for the gene pertussis toxin promoter region. An antigenic shift of *PtxP1* to PtxP3 was observed in all Iranian clinical isolates [50,51]. Reports of the ptxP3-type strains in India is consistent with these studies. The reason for the observation of ptxP3-type strains in India may be associated with the increase in the usage of acellular vaccines (aP) by the private market in India, or a lack of data on genome epidemiology of clinical isolates [52].

While DTwP vaccines include all antigens of *B. pertussis*, along with PT, PRN, FHA, and FIM 2 and 3, genetic change was apparently not a problem in the DTwP countries [53]. However, with the recent emergence and spread of a new Bordetella variant, genetic variation has become a major concern regarding vaccine efficacy [54,55]. Nowadays, the ptxP3 strains, reported to be more virulent as suggested by increased pertussis toxin production, indicate selective advantage under vaccine mediated selection pressure [56,57]. Therefore, current whole-cell and acellular vaccine efficacy should be evaluated against strains carrying the ptxP3 allele type [58]. India represents an excellent opportunity for such studies, as the Indian vaccine industry is the largest global supplier of wP, with more than seventy percent of the pentavalent combined vaccines global requirement is provided by vaccine manufacturing companies in India [59]. Characterization of vaccine strains from India and global comparison with *B. pertussis* diversity is equally important [60].

The protective efficacy of vaccines should be evaluated using different challenge models, using both in vitro and animal models [61,62]. The strains characterized in the animal model as well as by proteomics and transcriptomics with the closed genome available should be evaluated at a clinical level in human challenge models [63]. A similar study is reported using a *B. pertussis* isolate B1917, which is representative of circulating isolates in Europe [64]. The use of clinical isolates from the developing countries using wP vaccines in such studies will also play a significant role in determining the impact of differential vaccination and pathogen adaptation, which will be imperative in designing prevention strategies to control the resurgence of the disease.

A limitation of this study is the low sample size; however, this study reports a maximum number of samples and data on genomic characteristics of clinical isolates of *B. pertussis* reported from India so far. This study should provide a good basic for further examining of the circulating isolates and vaccine strains of *B. pertussis* from India and many developing countries using wP to study pathogen adaptation.

## 4. Materials and Methods

### 4.1. Sample Collection and Processing

We characterized five *B. pertussis* clinical isolates S1, S2, S3, S4 and S5 recovered from clinical samples collected from patients hospitalized or visited with clinical features such as pertussis visited or admitted in a pediatric hospital in the Pune region within India [65]. Nasopharyngeal swabs samples were received in Regan-Lowe transport medium (RTM) (Copan) under ambient conditions [26]. Detailed information of all clinical samples is included in Table 1. The swabs were streaked on Bordet-Gengou Agar (BGA, Difco) plates with defibrinated horse blood 15% (*v*/*v*) (in house) with cephalexin (40 µg/mL) (Oxoid, UK) to inhibit common Gram-positive nasopharyngeal flora in the sample [66]. The plates were incubated at 35 °C for 3–5 days. Suspected colonies were sub-cultured from mixed microbial growth observed on the same medium for 24–48 h. Suggestive *B. pertussis* colonies were confirmed using phenotypic and molecular identification techniques [26,27,65].

### 4.2. Bacterial Identification

*B. pertussis* isolates were confirmed by a combination of colony morphology, conventional biochemical methods such as oxidase, Gram staining abd growth characteristics. *B. pertussis* isolates were confirmed with agglutination observed with reference polyclonal *B. pertussis* specific immune sera (NIBSC). Cultures were further confirmed at genetic and protein level tools such as 16s ribosomal DNA sequencing using MicroSEQ™ Full Gene 16S rDNA PCR Kit (The MicroSeq, Applied Biosystems, Foster City, CA, USA) on a microbial identification system (The MicroSeq, Applied Biosystems, Foster City, CA, USA) and matrix-assisted laser desorption time of flight mass spectrometry (MALDI-TOF, Bruker, MA, USA) [27,28,41].

### 4.3. Serotyping

Serotype was determined by agglutination assay with monospecific antibodies for fim type 1 and type 2 (*Fim*2; NIBSC) and type 3 fimbriae (*Fim*3, NIBSC) antigens of *B. pertussis*, formerly called agglutinogens [18,19,29]. The bacterial suspension was prepared using 0.85% normal sterile saline solution. Briefly, 15 uL of bacterial suspension taken on a clean glass slide with antibodies against agglutinogen 1, 2, 3 (National Institute for Biological Standards and Control, NIBSC). *B. pertussis* vaccine strains were used as a positive control for serotype 1, 2 and 3. If the agglutination reaction was obtained with either agglutinogen 1, 2, 3 or both, the serotype was defined as 1,2 or 2,3, respectively. Agglutination was determined after 30 s to avoid a false positive reaction. Bacterial suspension was mixed with a normal saline to determine auto-agglutination. The deionized water was used as a negative control.

### 4.4. DNA Extraction and Multiplex PCR

Genomic DNA extraction from bacterial suspension was performed with QIAamp DNA Mini Kit (Qiagen, Redwood City, CA, USA) as per the manufacturer’s instruction. Total DNA concentration and purity were measured using NanoDrop^®^1000 (ThermoScientific, Waltham, MA, USA) and stored at −20 °C till further use. Multiplex standard polymerase chain reaction (PCR) was performed with oligonucleotide primers targeting genes to detect *B. pertussis*, *B. parapertussis* and *B. Bronchispetica* IS481, IS1001, IS1002, respectively, and virulence genes of *B. pertussis* (prn, *ptxA*, ptxP, cyaA, tcfA, FHA, fim2, *fim3*, dnt, ompQ, brkA, bapC, vag8). The housekeeping gene (pgm) of *B. pertussis* was also included in the target panel [64]. The sequences of the primers used are given in Supplementary Table S1. PCR reaction mixtures were prepared for 50 uL, containing 1.5 mM MgCl2, 0.2 uM of each forward and reverse primer, DNA, 10% DMSO (*v*/*v*) (Sigma-Aldrich, St. Louis, MO, USA), HotstarTaq Master Mix (Qiagen) containing 2.5 units HotstarTaq DNA Polymerase (1X) and 200 µM of each deoxynucleotides (dNTP) (Qiagen). PCR Reaction mixtures with no added template were used as the negative control. Amplification was performed in a thermal cycler (Eppendorf thermocycler, Nexus Flexid (Eppendorf AG, Germany) with cyclic conditions as: initial heat activation at 95 °C for 15 min, denaturation at 94 °C for 30 s, annealing at 64 °C for 30 s, extension at 72 °C for 1 min, followed by final extension at 72 °C for 10 min. Amplified DNA was observed using agarose gel electrophoresis (2%) (Sigma-Aldrich) stained with 0.5 ug/mL ethidium bromide (Sigma, Livonia, MI, USA). Gel was photographed using Gel Doc 2000 system (Bio-Rad, Hercules, CA, USA).

### 4.5. Genotyping

Polymorphism has been described in the pathogens genes encoding pertactin (*prn*), pertussis toxin (*ptxA*), pertussis toxin promoter (*ptxP*), fimbriae (*fim2*) and fimbriae (*fim3*). The standardized genotyping of these five genes was performed by DNA cycle sequencing [67]. The different alleles of the five genes previously described were used for allele identification. PCR amplification and sequencing were performed with the same forward, reverse primers used for initial PCR amplification of respective target gene. PCR products were further purified from agarose gel using QIAquick Gel extraction kit (Qiagen) as per the manufacture’s instructions. The purified gel elute was quantified using NanoDrop^®^1000 (ThermoScientific). PCR amplification was performed by adding 4 uL purified sample (template) to 6 uL of a Big Dye terminator reaction mixture containing 1 uL forward and reverse primer, respectively and 4 uL nuclease free water. Thermal cycling was performed with programme as 96 °C form 1 min, 25 cycles of 96° for 10s, 50 °C for 5s, 60 °C for 4 min and sequenced with an ABI-Prism Big Dye terminator reaction kit for ABI3500 Genetic analyzer (Applied Biosystem). Sequence analyses were performed using the software package Gene Base (V1.1). Sequence data from forward and reverse sequencing primers were combined and aligned using MEGA6 software [68]. Nucleotide sequence of the alleles in the prn, *ptxA*, ptxP, fim2 and *fim3* of clinical isolates S1, S2, S3, S4, S5 were compared with sequences in the GenBank database for different alleles of the respective gene.

### 4.6. Whole Genome Sequencing and Annotation

Whole genome sequences for all five isolates S1, S2, S3, S4 and S5 were obtained using a hybrid sequencing strategy combining long and short sequence reads generated using Oxford Nanopore (Long read) and Illumina (short read) sequencing [24]. For Illumina sequencing (Illumina, San Diego, CA, USA), bacterial DNA was extracted using Pure Link DNA isolation kit (Invitrogen™, Waltham, MA, USA) and visualized on an agarose gel (1%) and Qubit 4.0 fluorometer (Thermofisher #Q33238, Waltham, MA, USA) with DNA HS assay kit (Thermofisher #Q32851) according to the manufacturer’s instruction. Libraries were prepared using the Nextera XT DNA library preparation kit (Illumina, San Diego, CA, USA) and sequenced on a Novaseq 6000 platform (Illumina, San Diego, CA, USA) using (2 × 150 bp) chemistry. Genomic DNA was prepared for Nanopore sequencing by phenol chloroform extraction using the CTAB method and purified by ethanol precipitation. For Nanopores, the library was prepared using the native barcoding kit (EXP-NBD103, Oxford Nanopore Technologies, Oxford, UK) and 1D chemistry (SQK-LSK108).

### 4.7. Comparative Genomics

The genomes of all five isolates sequenced during this study were represented in circular graphical representation created using Circos [69]. For genome organization analysis, genomes of all 13, 6 vaccine reference strains and 7 *B. pertussis* isolates from India, were aligned using progressive Mauve software (version 2.0, Darling lab, University of Technology, Sydney, Australia) with default parameters [70]. All 13 *B. pertussis* genomes were compared to the 744 *B. pertussis* closed genome available in the public databases (as of 20 February 2021), including vaccine reference strains and 7 *B. pertussis* isolates from India using phylogenetic reconstruction from pairwise Mash (version 2.0) and distance using mashtree (version 0.29) [71].Tree was visualized and annotated using iTOL (v5, Leutnic I and Bork P) [72]. 

## 5. Conclusions

In conclusion, large-scale monitoring of genome epidemiology from countries using wP is imperative to study the pathogen adaptation and vaccine mediated selection pressure.

## Figures and Tables

**Table 1:** Demographic characteristics of *B. pertussis* isolates.

Sample	Place	Year	Age	Gender	Serotyping (*Fim*2/*Fim*3)	Genome Size	Accession No
S1	Pune	2018	3.5	Female	+/+	4124562	CP077657
S2	Pune	2019	3	Male	+/+	4102989	CP077656
S3	Pune	2019	13	Female	+/+	4109561	CP077655
S4	Pune	2020	02	Female	+/+	4109559	CP077654
S5	Pune	2020	1	Male	+/+	4135302	CP077653

**Table 2:** General genome features of *B. pertussis* genomes available from India.

Strain	Accession Number	Feature	ST	PtxP	*PtxA*	Prn	*Fim*3	*Fim*2
TohamaI	NC2099	Reference Strain	2	*PtxP1*	*PtxA*2	Prn1	*Fim* 3-1	*Fim* 2-1
J445	CP017402	Vaccine Strain	2	*PtxP1*	*PtxA*2	Prn1	*Fim* 3-1	*Fim* 2-1
J446	CP017403	Vaccine Strain	1	PtxP2	*PtxA*4	Prn7	*Fim* 3-1	*Fim* 2-2
J447	CP017404	Vaccine Strain	2	*PtxP1*	*PtxA*1	Prn1	*Fim* 3-1	*Fim* 2-1
J448	CP017405	Vaccine Strain	2	*PtxP1*	*PtxA*1	Prn1	*Fim* 3-1	*Fim* 2-1
Bp165	RSFF00001	Vaccine Strain	2	*PtxP1*	*PtxA*2	Prn1	*Fim* 3-1	*Fim* 2-1
Pelita III	CP019957	Vaccine Strain	1	*PtxP1*	*PtxA*2	Prn1	*Fim* 3-1	*Fim* 2-1
S1	CP077657	Clinical Isolate	2	*PtxP1*	*PtxA*2	Prn1	*Fim* 3-1	*Fim* 2-1
S2	CP077656	Clinical Isolate	1	*PtxP1*	*PtxA*2	Prn1	*Fim* 3-1	*Fim* 2-1
S3	CP077655	Clinical Isolate	2	PtxP3	*PtxA*2	Prn2	*Fim* 3-1	*Fim* 2-1
S4	CP077654	Clinical Isolate	2	PtxP3	*PtxA*2	Prn2	*Fim* 3-1	*Fim* 2-1
S5	CP077653	Clinical Isolate	2	*PtxP1*	*PtxA*2	Prn1	*Fim* 3-1	*Fim* 2-1
BPD1	CP034182	Clinical Isolate	2	*PtxP1*	*PtxA*1	Prn1	*Fim* 3-1	*Fim* 2-1
BPD2	CP034101	Clinical Isolate	2	*PtxP1*	*PtxA*1	Prn1	*Fim*3-1	*Fim*2-1

## Data Availability

All data generated or analyzed during this study are included in this article and supplementary information. Genomic data are deposited in GenBank databases under accession numbers (CP077657, CP077656, CP077655, CP077654).

## Supplementary Materials

The following supporting information can be downloaded at: https://www.mdpi.com/article/10.3390/pathogens11070794/s1, Table S1: Primers used for genotyping of *B. pertussis* strains.
