# Supplementary material for: Characterization of Bordetella pertussis Strains Isolated from India
Source: Pathogens. 2022 Jul 14;11(7):794. doi: 10.3390/pathogens11070794 (PMC9322502; doi:10.3390/pathogens11070794)
Supplement: Supplementary file 1 [file pathogens-11-00794-s001.zip › pathogens-1750960-supplementary.pdf]

Table S1. Primers used in the PCR amplification for identification of B. pertussis genes.

Gene	Primer Sequence
Prn	F: GCCAATGTCACGGTCCAA<br>R: CGGATTCAGGCGCAACTC
PtxP	F: AATCGTCCTGCTCAACCGCC<br>R: GGTATACGGTGGCGGGAGGA
Fim2	F: GCATAGACCATCTTGTATG<br>R: CCGGCCGGGCTCCTTGAG
Fim3	F: CCCCCGGACCTGATATTGTGATG<br>R: GCTGAGCGTGCTGAAGGACAAGAT
PtxS1	F: ACCATCAAAACGCAGAGGGGAAGA<br>R: AGGAGGGCCAACGGCAGA
cyaA	F: ACCGCCTACGGCAAGCGC<br>R: GCCGCCTTCAAGGGTATC
TcfA	F: ATGCACATTTACGGAAATATGA<br>R: TATGCGTGCCCGGGTCATAG
ompQ	F: ATGCGTCGTCTTCTCGTC<br>R: TCAGAAGCGCTGGGTCAT
BapC	F: GACAACGGTGTCTGGGGC<br>R: GCGCAGGTGGAACGTCCA
FHA	F: TCGCCATTTCGGCGCACG<br>R: AGATCGAGCTGCGCGCCG
Vag8	F: ATGGCAGGACAAGCGAGG<br>R: CCGCGTACCCGTCAACGT
Adk	F: AGCCGCCTTTCTCACCCAACACT<br>R: TGGGCCCAGGACGAGTAGT
BrkA	F: CCTGCTCGGCTACACCTATGC<br>R: CCTGGCGGGGTTTTTCATTG
IS481	F: GCCGGATGAACACCCATAAG<br>R: GCGATCAATTGCTGGACCAT
IS1001	F: AATTGCTGCAAGCCAACCA<br>R: CCAGAGCCGTTTGAGTTCGT
IS1002	F: CTAGGTCGAGCCCTTCTTGTTAAC<br>R: GCGGGCAAGCCACTTGTA
